# Estimating multivariate longitudinal trajectories using mixed-effects models with crossed random effects

**DOI:** 10.3758/s13428-026-03070-5

**Published:** 2026-06-17

**Authors:** José Ángel Martínez-Huertas, Emilio Ferrer

**Affiliations:** 1 Department of Methodology of Behavioral Sciences, National Distance Education University (UNED), Madrid, Spain; 2 Department of Psychology, University of California, Davis, CA USA

**Keywords:** Mixed-effects models, Crossed random effects, Variables, Continuous-time metric, Multivariate longitudinal data, Forecasting, Trajectories

## Abstract

In this study we examine how a mixed-effects model with crossed random effects for individuals and variables estimates within- and between-variability in longitudinal multivariate trajectories from cohort-sequential designs. These designs are characterized by large proportions of planned missing data, and they usually require continuous-time metrics. Via simulations, we evaluated different model outcomes under various conditions regarding the size of clusters (individuals and variables) and the complexity of the trajectories. Results show that (a) this model can estimate the general trajectories (common to all individuals and variables) and their variability, plus the variable-specific trajectories through the predictions of the levels of the random factors; (b) the standard errors of the random effects are wide, yet they are important for making substantive decisions for specific variables; and (c) the model predictions can adequately forecast individual and variable-specific complete trajectories from just a few observations per individual. These results are supported in an empirical illustration using cognitive developmental data. These findings show that researchers can obtain complete individual trajectories for multiple variables throughout a target age range. The relative simplicity of this model in comparison with other alternatives makes it a promising and accessible tool for multivariate longitudinal data analysis.

**Supplementary Information:**

The online version contains supplementary material available at 10.3758/s13428-026-03070-5.

## Introduction

**Fig. 1:**
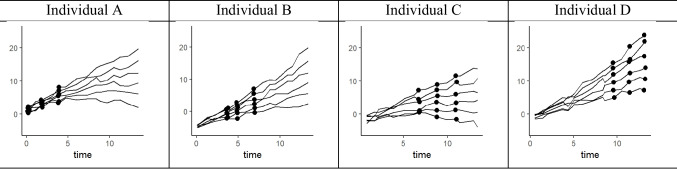
Variable-specific trajectories for six variables of four individuals from different cohorts of a hypothetical cohort-sequential design. *Note*. Dots represent the measurement of the variables (all are assumed to be measured at the same time point). Lines represent the target trajectory for the complete age range of the study for each variable

Different approaches have been developed for the study of multiple processes that change at the same time (McArdle, 2009). These statistical models usually aim to describe developmental trajectories of multiple variables, capturing intra-individual change and inter-individual differences in such change by means of latent variables. Very often, researchers are interested in capturing processes that span periods larger than the duration of a standard study. One common design used to accomplish this is the so-called cohort-sequential design (Bell, 1953, 1954). This design is efficient in capturing long developmental periods including participants contributing with only a few data points (e.g., Bell, 1953, 1954; Duncan et al., 1996; Estrada & Ferrer, 2019; Johal et al., 2021; McArdle, 1994; Miyazaki & Raudenbusch, 2000; Nesselroade & Baltes, 1979; Schaie, 1965; Voelkle & Hecht, 2020). Figure 1 presents hypothetical longitudinal data from six variables for four individuals in a cohort-sequential design where individuals are measured in three nonconsecutive years. In cohort-sequential designs, participants from different cohorts are repeatedly measured within a restricted age range, and their information is combined to represent a larger time period. As described in Fig. 1, the focus of these designs is to analyze the overall trajectory across the entire time range of the study, even though each individual only contributes with a few measurements. Moreover, in the case of multivariate longitudinal data, the main objective is to extend the analyses to further identify how multiple variables change at the same time in some individuals, capturing the common and the idiosyncratic variability of both variables and individuals.

In addition to presenting large proportions of missing data, cohort-sequential designs usually require a continuous-time metric, as individuals are measured at different occasions, and the intervals between such measurements typically vary across individuals (see Fig. 1). In other words, the observation times (and time intervals between them) are measured on a meaningful scale that can vary continuously, such as minutes, days, months, or years. Thus, time is included explicitly as a covariate in the model. This is different from, say, a continuous-time model (CTM), in which time is part of the outcome rate of change (e.g., Driver et al., 2017; Estrada & Ferrer, 2019; Ferrer et al., 2018; Martínez-Huertas et al., 2024; Martínez‐Huertas & Ferrer, 2026; Steele et al., 2018; Voelkle et al., 2012).

The use of a continuous-time metric presents substantive and methodological advantages relative to discrete time, although it usually requires more complex models such as the aforementioned CTM. In the case of univariate processes, multilevel modeling (aka mixed-effects or hierarchical linear models) has been widely used for assessing individual change in longitudinal data using discrete and continuous-time metrics since the original development of that approach (Bryk & Raudenbush, 1987). Multilevel models were shown to be suitable for capturing the mean trajectory of change in a sample as a combination of the initial level and the rate of change (using fixed effects), estimating individual variation around that mean trajectory (using random effects), estimating the correlation between the initial levels and the rate of change, assessing the reliability of measures for describing change (using the error term), examining whether some variables influence change (using additional covariates), and predicting future individual changes. These estimations are fundamental in longitudinal research and are the focus of latent variable approaches such as the popular latent growth curve models (McArdle, 2009).

This specification of the mixed-effects model is useful for the analysis of univariate longitudinal processes aimed at capturing a trajectory common to all individuals in a sample as well as their variation around it using a single random effect or cluster (see Raudenbush, 2001, for alternative specifications). In univariate systems, mixed-effects and latent growth curve models present a large, yet incomplete, mathematical and functional equivalence (McNeish & Matta, 2018). While there are more similarities than differences between the two approaches, some differences are worth noting (McNeish & Matta, 2018): (1) mixed-effects models require smaller sample size than structural equation models to estimate growth curves; (2) latent-basis models accommodate any nonlinear growth, but mixed-effects models are more flexible for testing specific nonlinear functions; and (3), important for the current paper, time-unstructured data are more easily specified in mixed-effects models than in structural equation models. In fact, data sparsity has been found to potentially affect structural equation models when the overlap between measurement occasions is small (McNeish & Matta, 2020; see also Martínez-Huertas & Ferrer, 2023, for differences in computing time with multivariate longitudinal analysis).

Analyzing univariate systems is useful for understanding the changes in some psychological phenomena, but the ultimate goal of many studies is to understand how multiple processes unfold over time. To accomplish this goal, multivariate models with latent variables are often the first choice (e.g., McArdle, 2009), but their complexity makes their implementation difficult, especially when time follows a continuous metric. Although there are continuous-time alternatives for the analysis of univariate or bivariate systems (e.g., Driver et al., 2017; Estrada & Ferrer, 2019; Ferrer et al., 2018; Martínez-Huertas et al., 2024; Steele et al., 2018; Voelkle et al., 2012), their extension to multivariate systems with three or more variables is not straightforward. One potentially useful approach for such multivariate data is the mixed-effects model with crossed random effects (MEM-CR) for individuals and variables (Martínez-Huertas & Ferrer, 2023). This is a relatively easy modeling approach that can include both discrete and continuous-time metrics.

**Fig. 2:**
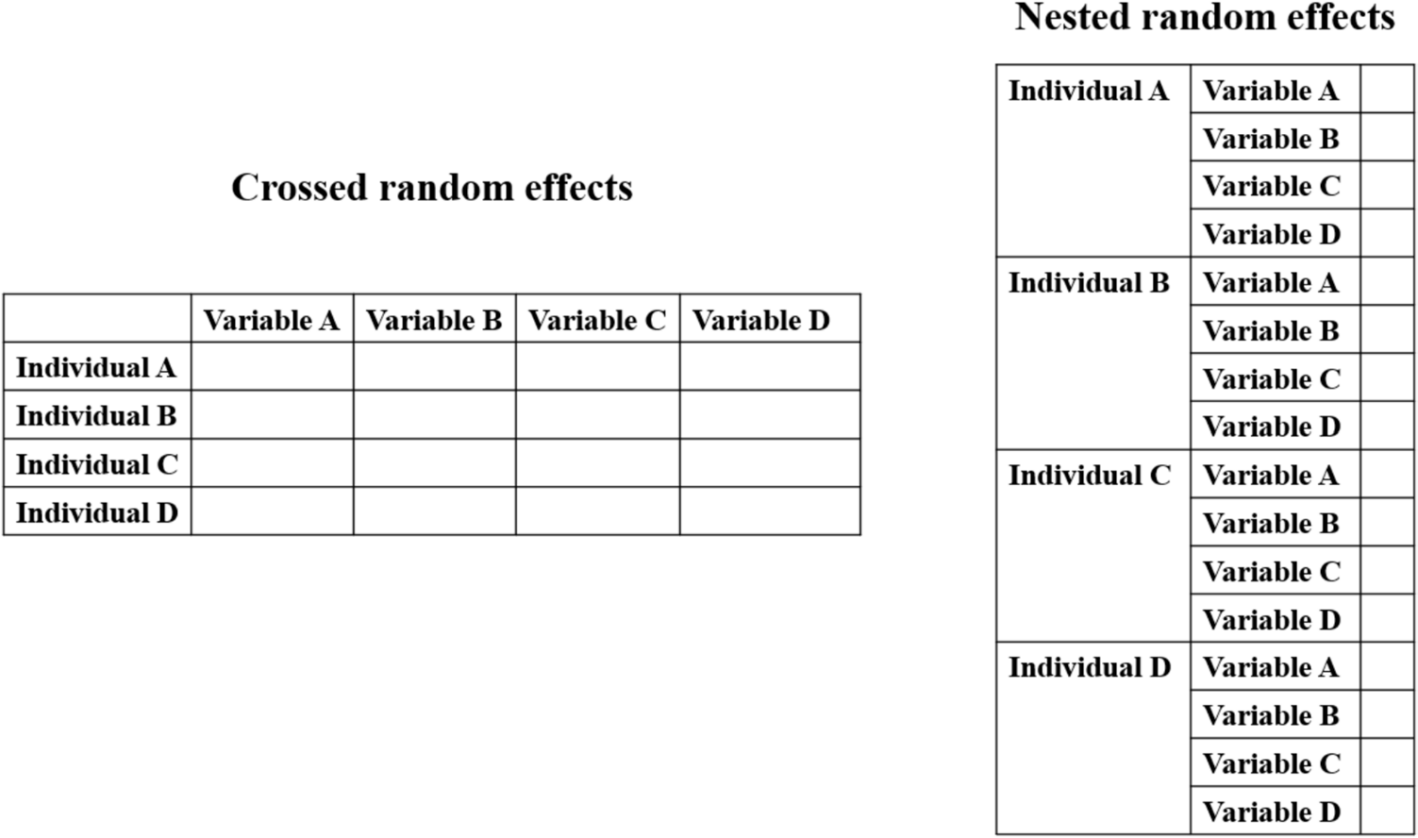
Graphical representation of the random effects comparing crossed and nested structures for four individuals and four variables. *Note*. Given that all the levels of the first random effect (individuals) are hypothetically measured in all the levels of the second random effect (variables), it is possible to accommodate crossed random effects for the analysis of multivariate longitudinal data. The assumption of this random structure is that all the levels of the clusters are crossed, although this procedure permits partial or complete missing combinations (i.e., it is not necessary to evaluate all individuals in all the variables to fit the model). The main advantage of crossed random effects, relative to nested random effects, is their flexibility for modeling nonhierarchical structures and handling multiple sources of variability at the same time

Although the MEM-CR model was proposed several decades ago, an efficient and fast estimation has not been possible until recently (e.g., Goldstein, 1987a, 1987b). This model has been used extensively in education (e.g., Garner & Raudenbush, 1991; Raudenbush, 1993), but popularized in psychology only recently (Baayen et al., 2008). In a typical specification, this model considers individuals and items as clusters at the same level, as opposed to nested within each other (see Baayen, 2008; Bates et al., 2015; Hoffman, 2015; Hoffman & Rovine, 2007; Quené & van den Bergh, 2008; see also Fig. 2). This popularization was favored by the development of the R *lme4* package (Bates et al., 2014). MEMs-CR are flexible in accommodating crossed random effects of individuals and variables (see Fig. 2), can handle missing data, and use a continuous-time metric, all important features of cohort-sequential designs (Martínez-Huertas & Ferrer, 2023). Despite this flexibility, however, there is no evidence on the performance of MEMs-CR regarding important data conditions such as the use of a continuous-time metric to analyze the multivariate change with large proportions of missing data (like the cohort-sequential design) for forecasting individual trajectories. In this paper, we address, this issue by evaluating the performance of the MEM-CR for individuals and variables to estimate general and variable-specific trajectories in cohort-sequential designs (for the study of univariate processes, see for example Raudenbush, 2001; Bryk & Raudenbush, 1987). In addition, we aim to identify reasonable inferences at both the individual and variable levels through the analysis of the standard errors of random effects and the predictions of individual scores. We also evaluate the performance of these models in forecasting individual scores for predicting both general and variable-specific trajectories.

### MEMs-CR for individuals and variables in multivariate longitudinal research

The MEM-CR was recently described as a promising tool for estimating growth curves in multivariate longitudinal data using likelihood-based estimations for both discrete- and continuous-time metrics^1^ (Martínez-Huertas & Ferrer, 2023). This model can describe curves with a large linear effect and a smaller negative quadratic effect, as trajectories typically observed in the development of cognitive abilities. The model can be expressed as follows:

1 $$\begin{array}{l}{y}_{ivt}=\left({\beta}_{0}+{u}_{0i}+{u}_{0v}\right)+\left({\beta}_{1}+{u}_{1i}+{u}_{1v}\right)ag{e}_{ivt}+\left({\beta}_{2}+{u}_{2i}+{u}_{2v}\right)ag{e}_{ivt}^{2}+{\epsilon}_{ivt}, \\ with \\ \begin{array}{l}{u}_{i}=\left[\begin{array}{l}{u}_{0i}\\ {u}_{1i}\\ {u}_{2i}\end{array}\right]\sim N\left(\left[\begin{array}{l}0\\ 0\\ 0\end{array}\right], {\Sigma}_{A}=\left[\begin{array}{ccc}{\sigma}_{0A}^{2}& {\sigma}_{01A}& {\sigma}_{02A}\\ {\sigma}_{01A}& {\sigma}_{1A}^{2}& {\sigma}_{12A}\\ {\sigma}_{02A}& {\sigma}_{12A}& {\sigma}_{2A}^{2}\end{array}\right]\right), \\ {u}_{v}=\left[\begin{array}{c}{u}_{0v}\\ {u}_{1v}\\ {u}_{2v}\end{array}\right]\sim N\left(\left[\begin{array}{c}0\\ 0\\ 0\end{array}\right], {\Sigma}_{B}=\left[\begin{array}{ccc}{\sigma}_{0B}^{2}& {\sigma}_{01B}& {\sigma}_{02B}\\ {\sigma}_{01B}& {\sigma}_{1B}^{2}& {\sigma}_{12B}\\ {\sigma}_{02B}& {\sigma}_{12B}& {\sigma}_{2B}^{2}\end{array}\right]\right), \; and \\ {\epsilon}_{ivt}\sim N\left(0, {\sigma}_{\epsilon }^{2} \right),\end{array}\end{array}$$

where ${y}_{ivt}$ is the dependent variable score of individual *i* for variable *v* at time *t*, ${\beta}_{0}$ is the intercept or initial level (common to all individuals and variables) at time *t* = 0 (in this study, the age at the first measurement occasion), ${\beta}_{1}$ is the average linear effect of time (common to all individuals and variables), ${\beta}_{2}$ is the average quadratic effect of time (common to all individuals and variables), and ${\epsilon}_{ivt}$ is the error term for individual *i* and variable *v* at time *t*, with its own error variance. Random effects, ${u}_{i}$ and ${u}_{v}$, are defined with a normal distribution with zero mean and unknown standard deviation around a specific fixed effect, which are the cluster means (e.g., Baayen et al., 2008; Bates et al., 2014, 2015; Hoffman, 2015). Among other possibilities, the estimation of this model allows us to study the general (common to all individuals and variables), individual-specific (common to all variables), variable-specific (common to all individuals), and both individual- and variable-specific trajectories by means of crossed random effects (see Fig. 2).

The underlying assumption of this model is that there is a common developmental process for all individuals and variables (or, at least, that their development tends to covary), and it is possible to predict specific trajectories for them, which will depend on how close the individuals or the variables are to those common time effects. In sum, these models allow us to estimate (1) the general longitudinal trajectories via the fixed effects, which represent the population-level estimations for all individuals and variables, and (2) the variable-specific (and individual-specific) trajectories, as a function of the variability of the levels of the clusters (variables and/or individuals). To accomplish this, it is helpful to standardize the variables based on the corresponding first measurement occasion for analyzing these multivariate longitudinal trajectories. This is especially important regarding the interpretability of the parameters (both fixed and random effects and best linear unbiased predictions [BLUPs], details below) in relation to the cluster of variables.^2^

Randomly sampling a set of individuals from a specific population would ideally lead to a representative sample that will describe the general (common to all individuals) longitudinal trajectory by means of fixed effects. This is a strong but generally accepted assumption in almost all empirical studies in psychology (cf. Molenaar, 2004), typically known as the exchangeability principle in hierarchical linear models (Lindley & Smith, 1972; Raudenbush, 1993; see also Draper et al., 1993; Greenland & Draper, 2005). The same rationale applies to the variable-specific trajectories generated from the use of variables as a random effect. One researcher could randomly sample a set of variables of a more general psychological construct, which would ideally lead to a representative sample of variables that will describe the general (common to all variables) longitudinal trajectory. This may seem to be a stronger assumption, but it clearly aligns with many theoretical models that assume that there are latent variables underlying different observations, also known as common factor models. In other words, variables are treated as indicators of the same underlying construct. Under a classic perspective, discrepancies among them may be interpreted as random measurement error rather than substantive differences. But that kind of measurement error should be captured in the error term, and the random effects are supposed to capture substantive variability regarding the intercepts and slopes of both individuals and variables.

The parametrization of the MEM-CR for individuals and variables could be understood as a blend of composite and common factor models (Rhemtulla et al., 2020). Thus, using the MEM-CR for individuals and variables would imply, for example, that one is able to measure different cognitive functions (e.g., memory, language, attention) that share a common development, or, at least, that their developmental trends covary. As such, applying this model assumes exchangeability for the different levels of such random effects (e.g., Hoffman, 2015; Raudenbush, 1993). Thus, this approach is not reasonable for just any set of variables that more or less measure similar constructs, but it should be applied to variables that are “exchangeable” with respect to the underlying construct they are meant to measure. Such exchangeability should be evaluated in the changes of individuals and variables included in the model more than in the variables themselves measured in the individuals at a particular time. For instance, in the study of cognitive abilities, this approach would require some theoretical assumptions regarding common developmental processes (e.g., McArdle, 2009; McArdle et al., 2002), which may implicitly assume the existence of common factors (e.g., Colom et al., 2006; Garlick, 2002; Humphreys, 1979).

It is known that the intercept and slope of individuals and variables can diverge considerably from the population means (see Molenaar, 2004, for a theoretical rationale). That is, even when the estimated trajectory of the fixed effects shows a linear change, there can be individuals and variables with no change or even with negative changes. This idiosyncratic variability of individuals and variables can be captured by random effects. Such estimations of the different levels of a random factor in a specific fixed effect are referred to as best linear unbiased predictions (BLUPs; see for example Baayen, 2008; Henderson, 1975; Robinson, 1991). Once the parameters ($\widehat{{\boldsymbol{\theta}}}$) are estimated using a model like the one from Eq. 1, the marginal covariance of the data is obtained as $V={Z}_{i}{\widehat{\Sigma }}_{A}{Z}_{i}^{T}+{Z}_{v}{\widehat{\Sigma }}_{B}{Z}_{v}^{T}+{\widehat{\sigma }}_{e}^{2}I$, where ${Z}_{i}$ and ${Z}_{v}$ define the effects for each level of the clusters as a block-diagonal matrix. Then, given a set of estimated fixed effects ($\widehat{{\boldsymbol{\beta}}}$), the BLUPs of the crossed random effects can be expressed as

2 $$\begin{array}{c}{\widehat{{\boldsymbol{u}}}}_{i}={\widehat{\Sigma }}_{A}{Z}_{i}^{T}{V}^{-1}\left({\boldsymbol{y}}-{\boldsymbol{X}}\widehat{{\boldsymbol{\beta}}}\right) \;\mathrm{and} \\ \,{\widehat{{\boldsymbol{u}}}}_{v}={\widehat{\Sigma }}_{B}{Z}_{v}^{T}{V}^{-1}\left({\boldsymbol{y}}-{\boldsymbol{X}}\widehat{{\boldsymbol{\beta}}}\right),\end{array}$$

which combine the data and the estimated variance components to obtain the most probable random‐effect values (i.e., the values with the lowest least squares). If the estimated ${\widehat{\Sigma }}_{A}$ and ${\widehat{\Sigma }}_{B}$ matrices are treated as known, it is possible to obtain a posterior covariance matrix for each level of both random effects via empirical Bayes, i.e., $Cov({{\boldsymbol{u}}}_{{\boldsymbol{i}}}|{\boldsymbol{y}})$ and $Cov({{\boldsymbol{u}}}_{{\boldsymbol{v}}}|{\boldsymbol{y}})$.

A relatively underexplored topic in the literature is the study of the standard errors of the levels of random effects (see some exceptions in different contexts and models, e.g., Austin & Leckie, 2020; Carpenter et al., 2003; Verbeke & Lesaffre, 1997). In practice, it is possible to obtain the uncertainty of such predictions in terms of confidence intervals. Once we have the BLUPs from crossed random effects, ${\widehat{{\boldsymbol{u}}}}_{i}$ and ${\widehat{{\boldsymbol{u}}}}_{v}$, it is possible to estimate their standard errors as the square root of the aforementioned conditional variances (which are the diagonal of the “postVar” matrices): $Cov({{\boldsymbol{u}}}_{{\boldsymbol{i}}}|{\boldsymbol{y}})$ and $Cov({{\boldsymbol{u}}}_{{\boldsymbol{v}}}|{\boldsymbol{y}})$ (Gelman & Hill, 2006; Gelman et al., 2015). Then it is possible to express the standard errors of each BLUP with *j* levels ${\ell}$ (where *j* = 1, 2, …, *n*) as

3 $$\begin{array}{c}se\left({\widehat{u}}_{{ji}_{\left({\ell}\right)}}\right)=\sqrt{{\left[Cov\left({\widehat{u}}_{{i}_{\left({\ell}\right)}}\left|{\boldsymbol{y}}\right.\right)\right]}_{jj}} \;\mathrm{a}\mathrm{n}\mathrm{d}\\ se\left({\widehat{u}}_{{jv}_{\left({\ell}\right)}}\right)=\sqrt{{\left[Cov\left({\widehat{u}}_{{v}_{\left({\ell}\right)}}\left|{\boldsymbol{y}}\right.\right)\right]}_{jj}} \end{array}$$

Such standard errors will facilitate the generation of empirical-Bayes credible intervals (Gelman & Hill, 2006), which should provide approximate conditional frequentist coverage under normality conditions. Note that to obtain a real frequentist confidence interval, one would need to estimate the uncertainty of the variance parameters using, for example, parametric bootstrap (e.g., Austin & Leckie, 2020; Carpenter et al., 2003).

BLUPs can follow different scenarios (Baayen, 2008): (1) all the levels of the random factor share the same positive or negative sign, with the effect being generalizable and likely statistically significant; (2) the majority of the levels of the random factor share the same positive or negative sign of the fixed effect while others do not, with the effect partially generalizable and likely statistically significant; and (3) approximately the same number of levels of the random factor have the same and different sign as the fixed effect, with the effect not generalizable and likely not statistically significant. All these scenarios provide relevant information about the development of individuals and variables. Independently of the estimate of the fixed effect and its statistical significance, such predictions provide relevant information regarding the individual-specific and variable-specific trajectories. For example, in a multivariate longitudinal study, a quadratic effect of time might not achieve statistical significance as a matter of statistical power if half of the variables show a pronounced quadratic effect while the other half show a linear trajectory (Martínez-Huertas & Ferrer, 2023).

These ideas agree with previous proposals about the use of random effects as a confirmatory hypothesis approach (e.g., Barr, 2013; Martínez-Huertas & Olmos, 2022). In the context of longitudinal studies, we believe that these systematic variations could be incorporated into the theories about the development of psychological constructs. Here, MEMs-CR for individuals and variables arise as a new opportunity to formalize such systematic variations around fixed effects using a relatively simple model compared with other alternatives that may require some constraints to converge or need significantly greater computation time (Martínez-Huertas & Ferrer, 2023). These previous results could be explained by the flexibility of the MEMs-CR for accommodating time-unstructured data in multivariate systems, as compared to structural equation models (see also McNeish & Matta, 2018, 2020, for the case of univariate systems).

### The present study

Previous research has shown that it is possible to extract information about within- and between-variability of development from MEMs-CR for individuals and variables similar to some latent variable approaches (Martínez-Huertas & Ferrer, 2023). However, to our knowledge, no previous study has analyzed the estimation performance of this model for recovering the generating process in cohort-sequential designs. This task is not straightforward, as it requires estimating parameters using a continuous-time metric to capture the longitudinal change in multivariate systems under conditions with large proportions of inherent missing data for forecasting individual trajectories in multiple variables. That said, the aim of the present study is threefold. We attempt to accomplish these goals with simulations and an empirical illustration.

Our *first goal* is to evaluate the performance of the MEM-CR in estimating a general longitudinal trajectory (common to all individuals and variables) and its variability across individuals and variables. In other words, the goal is to determine whether the fixed effects and their variance are correctly estimated in scenarios with multivariate growth curves of different complexity (linear, quadratic, and mixed). The estimation of these parameters is crucial for describing change and its variability across individuals and variables. Similarly, we evaluate the performance of the model in predicting the variable-specific trajectories. In particular, we focus on the recovery of predictions of the levels of the random factors, also known as variable-specific. Apart from describing the general trend common to all individuals and variables of the study, we examine whether the variable-specific predictions of the levels of the variables are accurate in describing the trends of the variable-specific trajectories. While this objective may be seen as simple, it is not straightforward to recover the population parameters and to adequately partition the observed variability when researchers are sampling just a few observations per individual and variable that also have measurement error. Specifying and estimating these unobserved components is a challenging task even in less demanding conditions (Grilli & Rampichini, 2015).

Our *second goal* is to evaluate the performance of the standard errors of the random effects to determine whether the model is useful for making decisions at both the individual and variable levels. Quantifying uncertainty in point estimates is important, and standard errors are a simple way of obtaining confidence intervals with known coverage probability (at least in a frequentist sense, although technically our approach uses empirical-Bayes credible intervals). Thus, this goal has important substantive implications and, to our knowledge, has not been examined, either for random-effect estimates or for continuous-time metrics in cohort-sequential designs. Previous research has analyzed the standard errors of fixed effects of the MEM-CR (e.g., Martínez-Huertas et al., 2022), but the standard errors of random effects are qualitatively different. In particular, we aim to analyze the accuracy of both the predictions of the levels of random effects and their standard errors in order to make inferences for each of the levels of the clusters of the model (in our case, with a special focus on the variables of the multivariate system). As we will show, this information is substantively rich and is derived directly from the random effects, although previous research did not give it much prominence (some exceptions include Austin & Leckie, 2020; Carpenter et al., 2003; Verbeke & Lesaffre, 1997). Previous research on multilevel models has devoted much more attention to the standard errors of fixed effects, as they are usually the main target of the analysis, which has led researchers to largely ignore the potential of predictions of random effects.

Our *third goal* is to examine whether the MEM-CR for individuals and variables can adequately predict individual and variable-specific trajectories from a few observations per individual. Our aim is to evaluate the model predictions for individual trajectories of the multivariate dataset for the complete age range under study, based on the age-restricted observations available for each individual. This would allow one to forecast both past and future points for specific cohorts of the study for each variable of the multivariate system. For example, one could predict future states of the individuals and variables in younger cohorts (e.g., predicting the scores at 15–19 years when individuals were measured at 5–9 years) or past states in older cohorts (e.g., estimating individuals’ scores at ages 5–9 years when they were measured at ages 15–19). Additionally, if the model yields precise predictions for individual and variable-specific trajectories, this may be interpreted as complementary evidence of the validity of the model estimates.

## Methods: Simulation study

### Simulated parameters

The parameter values used in the present simulation were based on the implementation of an MEM-CR (Martínez-Huertas & Ferrer, 2023) using a US cohort-sequential design, the National Longitudinal Survey of Youth 1979 (NLSY79) data (Center for Human Resource Research, 2009; Chase-Lansdale et al., 1991). We refer readers to this previous work to understand the selection of the values used in this simulation study. As reported in Martínez-Huertas and Ferrer (2023), the common developmental growth of different cognitive functions (reading comprehension, memory for digit span backward, memory for digit span forward, mathematics, vocabulary, and reading recognition) in individuals from 3 to 21 years was characterized by a large linear effect and a smaller negative quadratic effect. In that MEM-CR, a large positive linear effect and a small negative quadratic effect of age were found to describe the development of such cognitive abilities. Among other relevant findings, the estimates of the model showed that some of the variables had mainly linear trajectories, while others had important quadratic effects.

In the current project, we simulated a cohort-sequential design whose age range covered 15 years (e.g., from 5 to 19 years) over a period of 6 years (Table 1; see also Estrada & Ferrer, 2019). We considered two different sample sizes: 200 and 500 individuals. Each of the individuals was measured in all the variables in three nonconsecutive years. To simulate these data, we considered approximately one measurement per week (52 measurements per year) and then randomly selected one of the observations per year and individual (i.e., one individual could be measured at 5.19 years while other could be measured at 5.89). This made a total of 15 observations per individual. From these 15 observations, we randomly selected three observations per individual in nonconsecutive years following the cohorts described in Table 1. Each of these measurements were made for 6, 10, or 20 variables, depending on the simulation condition.

**Table 1:**
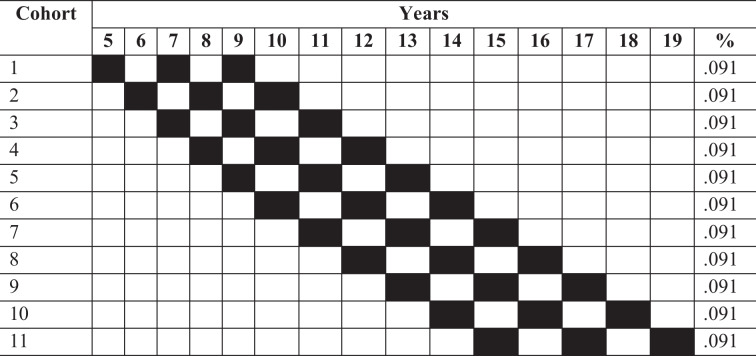
Simulated cohort-sequential design with individual measurements of all variables in three nonconsecutive years

Black cells represent a measurement per individual. % represents the approximate percentage of individuals in each cohort of the study (i.e., we simulated the same number of individuals in each cohort in this case)

We chose six variables as a representative example of multivariate longitudinal studies where the objective is to study the common development of a relatively small number of variables. For example, results from Martínez-Huertas and Ferrer (2023) represent this scenario. In the case with the most variables, 20 were chosen as an example of the number of items of a scale. For example, a multivariate longitudinal study may be focused on analyzing the common development of multiple items of, say, mathematics or vocabulary, and estimating their differences as random effects. This scenario is similar to that included in the original proposal of this model in psychology (e.g., Baayen et al., 2008) and is in line with work evaluating reliability in longitudinal data (e.g., Castro-Alvarez et al., 2025; Nezlek, 2017). In this kind of study, our perspective introduces additional substantive interest in the estimations of items as random effects.

Given a specific number of individuals (*n*_i_ = 200, 500) and variables (*n*_v_ = 6, 10, 20), three different scenarios were simulated, depending on the type of variable-specific trajectories: linear, quadratic, or mixed (both linear and quadratic). These conditions cover trajectories of different complexity, ranging from easily predictable linear trajectories to more complex ones. In all conditions, the general intercept (grand mean for *t* = 0) was set to − 2, and the linear fixed effect was 1. The quadratic fixed effect was set to −.025 in variables with quadratic effects in the two last simulation scenarios (i.e., it was fixed to zero for variables with linear trajectories). These values represent the general longitudinal trajectory common to all the variables included in the study.

In addition to the fixed effects, different random variances were established around those fixed effects generating variability for the intercepts and the slopes of both individuals and variables. For individuals, the standard deviation of random intercepts was fixed to 1, for linear effects it was fixed to.15, and it was fixed to.025 for random quadratic effects. In particular, the random coefficients of the variable-specific trajectories were defined by intercepts varying from −.50 to.50, linear effects varying from −.25 to.25, and quadratic effects varying from −.025 to.025. We selected the random coefficients of the variables to study the recovery of their point estimates, and thus correlations were not fixed. The uncontrolled variability of the observations in this simulation was random error associated with measurement errors, with the random standard deviation of error equal to.50 (i.e., approximately, 22–23% of the variance of each observation was measurement error), which differentially affected all the observations of the different individuals and variables sampled in the study.

Table 2 presents the simulation conditions of the present study. To summarize, we considered three general simulation scenarios by generating variable-specific trajectories that were all linear, all quadratic, or mixed (half linear and half quadratic). We included different numbers of individuals (*n*_i_ = 200 vs. 500) and variables (*n*_v_ = 6 vs. 10 vs. 20) to evaluate the minimum sample sizes required to recover the simulated parameters. We ran 1,000 replications for each of the 18 conditions.

**Table 2:** Conditions of the present simulation study

Manipulated conditions	Trajectories	Linear vs. quadratic vs. mixed
	Number of individuals (*n*_i_)	200 vs. 500
	Number of variables (*n*_v_)	6 vs. 10 vs. 20
Fixed conditions	Fixed intercept or grand mean (${\beta}_{0}$)	− 2
	Fixed linear slope (${\beta}_{1}$)	1
	Fixed quadratic slope (${\beta}_{2}$)	−.025
	Random intercepts, linear and quadratic effects – individuals (${\sigma}_{0A}$, ${\sigma}_{1A}$, ${\sigma}_{2A}$)	1,.15,.025
	Random intercepts – variables (BLUPs)	From −.50 to.50
	Random linear slope – variables (BLUPs)	From −.25 to.25
	Random quadratic slope – variables (BLUPs)	From −.025 to.025
	Random error (${\epsilon}_{ivt}$)	.50

The random effects of individuals and the random error are expressed in standard deviations for interpretability. The random effects of variables are expressed as BLUPs (see the presentation of Eq. 2)

### Data analysis

Each replication was analyzed using a MEM-CR with a continuous-time metric using the R *lme4* package (Bates et al., 2014). These models included random intercepts and random slopes for individuals and variables. For the condition of linear variable-specific trajectories, the model only included a linear effect of time. In both the quadratic and mixed conditions, the model included both linear and quadratic effects of time. Correlations between random effects were freely estimated. The R *arm* package (Gelman & Hill, 2006; Gelman et al., 2015) was used to estimate the standard errors of random effects. R code is provided in the supplementary materials to illustrate the implementation of these models. A restricted maximum likelihood (REML) estimator was used to fit the models as previously recommended in similar models (e.g., Hoffman, 2015; Martínez-Huertas et al., 2022) and similar contexts (e.g., Jiang, 1996; Thompson, 1962; Vasdekis & Vlachonikolis, 2005). As described in Hoffman (2015), differences can be expected between the likelihood of maximum likelihood and REML estimators, with REML specifically recommended for the estimation of random effects.

Once the models were fitted to each replication, we extracted the estimations of the fixed (intercept, linear, and quadratic effects of time) and random effects (random variances, predictions of the variable-specific trajectories, and their standard errors). In addition, we computed the predictions of the model for all the observations that were simulated, for each individual and variable. That is, we predicted the complete trajectories with the 15 theoretical measurements per individual and variable. We separated these predictions into those of the three available observations per individual and variable, and those for the remaining 12 unobserved scores per individual and variable that completed the simulated longitudinal trajectory. These analyses aimed to study the recovery of the original observed scores per individual and variable, as well as to determine whether the model could adequately forecast the remaining unobserved scores based on the available observations, for each individual and each variable.

To evaluate the performance of the model, we computed different indicators. To address our first goal (estimating the general trajectories and their variability), we computed bias and percentage of bias. For the estimations of the fixed effects and their variance, we computed bias using the following formula: $bias={\uptheta}_{est}-\theta$, where ${\uptheta}_{est}$ is the estimated parameter in each replication and θ is the simulated population parameter. The percentage of relative bias (RB) was computed as $RB=\left(\frac{{\uptheta}_{est}-\theta }{\theta }\right)\times 100$. RB was considered adequate when it ranged from − 10% to 10%. For the predictions of the levels of random effects (that is, the variable-specific trajectories), we computed the root mean square error (RMSE) of the difference between the simulated and the predicted coefficients of the random effects for variable-specific trajectories. We calculated the RMSE as $RMS{E}_{v}^{(k)}=\sqrt{\frac{1}{n}{{\sum}_{i=1}^{n}\left({\widehat{\beta }}_{iv}^{(k)}-{\beta}_{iv}\right)}^{2}}$, where ${\widehat{\beta }}_{iv}^{(k)}$ is the predicted coefficient (intercept, linear, and quadratic slopes) for variable *v* and individual *i* in replicate *k*, and ${\beta}_{iv}$ is the corresponding simulated coefficient.

For the second goal (evaluating the standard errors of the random effects), we computed coverage and overlap. Coverage was specified as the proportion of replications where the true simulated parameter of the levels of random effects was included in the 95% empirical-Bayes credible intervals constructed by the standard errors of the random effects. Given the conditions of different number of variables (i.e., 6, 10, and 20 variables), coverage was computed as the mean coverage for the different predictions of the levels of random effects of each replication. Overlap was computed as the proportion of comparisons of empirical-Bayes credible intervals around the estimates of the levels of random effects that intersect. Thus, it can be interpreted as a measure of the precision of the standard errors of the random effects. We computed the 95% empirical-Bayes credible intervals around the predictions for each of the levels of random effects of the variables, and then evaluated the overlap between them for the different variables. In particular, this was computed as the mean overlap due to the different numbers of variables (i.e., 6, 10, and 20 variables). For example, we evaluated the mean overlap using 15 comparisons of empirical-Bayes credible intervals in the case of six variables, and 190 comparisons in the case of 20 variables.^3^

Finally, to address our third goal (forecasting trajectories for observed and unobserved individual scores), we used RMSE. This was computed as the difference between the predicted individual scores for variable-specific trajectories and the true simulated trajectories as $RMS{E}_{iv}^{(k)}=\sqrt{\frac{1}{T}{{\sum}_{t=1}^{T}\left({\widehat{Y}}_{ivt}-{Y}_{ivt}\right)}^{2}}$, where ${\widehat{Y}}_{ivt}$ represents the predicted scores of individual *i* for variable *v* at measurement *t*, ${Y}_{ivt}$ represents the true scores of the simulated trajectories, and *T* is the number of time points. We computed two different versions of RMSE: one for the three observed individual scores per variable, and another for the 12 unobserved (but forecasted) individual scores per variable. Additionally, we computed the mean bias of the difference between the predicted scores of individual *i* for variable *v* at measurement *t* and the simulated true score in the 12 unobserved (but forecasted) individual scores to study potential shrinkage effects. To compute this bias, we used this formula for each individual: $bia{s}_{i}=\frac{1}{k}{\sum}_{i=1}^{k}({\widehat{Y}}_{ivt}-{Y}_{ivt})$, where *k* is the number of unobserved (but forecasted) individual scores per variable (*k* = 12), and then we computed the mean for $bia{s}_{i}$ in each cohort depending on the variable. As previously noted, these results can also be interpreted as additional evidence of the validity of the model estimates.

## Results

### Bias and percentage of bias of fixed and random effects of time

Table 3 presents the bias and the percentage of bias of the fixed effects and their variance as a measure of the model performance for estimating the general trajectories and their variability. We found that bias was almost zero for all the parameters of the model, regardless of the simulation conditions (linear, quadratic, or mixed trajectories), the number of individuals, variables, and parameters. The same applies to the percentage of bias, although larger values can be observed for the estimated variance of the variable-specific trajectories. This is probably due to a distortion associated with the standardization of bias, which divides by small simulated values, and reporting the mean percentage of bias instead of the median. Some authors have argued that relative bias smaller than ± 30% would be acceptable for variance parameters in multilevel models with random slopes (Grund et al., 2016; see also Speidel et al., 2018). Even with that lenient criterion, we would find overestimation of linear slopes of variables in quadratic trajectories for larger sample sizes in the individuals cluster when considering the mean of the percentage of bias. Despite this, the predictions of the model for trajectories of both individuals and variables are very accurate. It is worth noting that the median percentage of bias was much more adequate for all the parameters of the model, thus showing an asymmetric distribution of percentage of bias where some replications presented extreme values that distorted the mean.

**Table 3:** Bias and percentage of bias (%) of the estimations of the fixed effects and their variance within the crossed random effects for individuals and variables

	No. individuals	200						500					
	No. variables	6		10		20		6		10		20	
		Bias	%	Bias	%	Bias	%	Bias	%	Bias	%	Bias	%
Linear trajectories	Fixed intercept	.00	.15	.00	−.08	.00	−.09	.00	.05	.00	−.01	.00	.03
	Fixed linear slope	.00	.03	.00	.01	.00	.01	.00	−.02	.00	−.03	.00	−.03
	Random intercepts—individuals	.00	−.42	.00	−.43	.00	−.18	.00	.01	.00	−.01	.00	−.14
	Random linear effects—individuals	.00	−.21	.00	.06	.00	−.21	.00	−.03	.00	−.02	.00	−.12
	Random intercepts—variables	.00	−.01	.00	.18	.00	.24	.00	.02	.00	.31	.00	−.02
	Random linear effects—variables	.00	.08	.00	.00	.00	−.03	.00	.00	.00	−.07	.00	.02
Quadratic trajectories	Fixed intercept	.00	−.06	.00	−.22	.00	.20	.00	−.23	.00	.15	.00	.07
	Fixed linear effect	.00	−.03	.00	−.17	.00	.03	.00	−.06	.00	.09	.00	.04
	Fixed quadratic effect	.00	.26	.00	.41	.00	−.05	.00	.35	.00	−.19	.00	.06
	Random intercepts—individuals	.00	.01	.00	−.37	−.01	− 1.09	.00	−.18	.00	−.34	.00	.41
	Random linear effects—individuals	.00	−.74	.00	− 2.53	.00	− 2.50	.00	− 2.76	−.01	− 3.44	.00	− 1.71
	Random quadratic effects—individuals	.00	.63	.00	−.07	.00	−.45	.00	−.03	.00	−.75	.00	−.07
	Random intercepts—variables	.02	6.25	.02	5.31	.01	3.06	.04	11.99	.05	14.04	.05	15.50
	Random linear effects—variables	.02	8.21	.01	5.72	.01	4.05	.06	29.95	.06	32.84	.06	35.82
	Random quadratic effects—variables	.00	−.22	.00	4.20	.00	2.87	.00	15.20	.00	15.50	.00	16.84
Mixed trajectories	Fixed intercept	.00	−.06	.00	−.22	.00	.21	.00	−.24	.00	.15	.00	.07
	Fixed linear effect	.00	−.03	.00	−.16	.00	.03	.00	−.06	.00	.09	.00	.04
	Fixed quadratic effect	.00	.27	.00	.40	.00	−.07	.00	.35	.00	−.19	.00	.07
	Random intercepts—individuals	.00	−.03	.00	−.22	.00	−.43	.00	−.39	−.01	−.64	.00	−.03
	Random linear effects—individuals	.00	−.36	.00	− 1.51	.00	− 1.20	.00	− 2.21	.00	− 2.72	.00	− 1.20
	Random quadratic effects—individuals	.00	.73	.00	.21	.00	−.09	.00	.07	.00	−.61	.00	−.02
	Random intercepts—variables	.01	1.39	.00	.88	.00	.25	.00	1.22	.00	.27	.00	.07
	Random linear effects—variables	.00	.11	.00	.21	.00	.27	.00	.19	.00	.19	.00	.23
	Random quadratic effects—variables	.00	.25	.00	.00	.00	.01	.00	.43	.00	.11	.00	−.03

Gray cells = large bias according to % of bias (acceptable is ± 10%). SD = standard deviation. Ind. = individuals. Var. = variables

## Root mean square error (RMSE) of predictions of the levels of random effects (variable-specific trajectories)

In order to evaluate the performance of the model in predicting the variable-specific trajectories, we computed the RMSE of the difference between the simulated and the predicted coefficients of the random effects. Table 4 includes the results of these analyses. Across all conditions, the RMSE values were very small, indicating that the predictions of the levels of the random factors were accurate. That is, the variable-specific intercepts and both linear and quadratic slopes can be adequately recovered by the model. In other words, the model adequately identified the point estimations of the variable-specific trajectories just from three observations per individual. That said, some small differences in RMSE can be seen in Table 4. First, slightly larger RMSEs were found for the intercepts, probably due to the larger variability of the random effects of this parameter. Second, increasing the number of individuals from 200 to 500 reduced RMSE, especially in the more complex trajectory settings (quadratic and mixed). The same applies to increasing the number of variables, suggesting that more information leads to better estimates. Third, the small differences between the complexity of the trajectories can be observed mainly in the intercepts, with the trajectories easier to estimate from linear models than from models with quadratic or mixed effects.

**Table 4:** Means (and standard deviations) of the root mean square error (RMSE) of the difference between the simulated and predicted coefficients of the random effects for variable-specific trajectories

Trajectories	No. individuals	200			500		
	No. variables	6	10	20	6	10	20
Linear	Intercepts	.02 (.01)	.02 (.01)	.01 (.01)	.01 (.01)	.01 (.01)	.01 (.01)
	Linear slopes	.00 (.00)	.00 (.00)	.00 (.00)	.00 (.00)	.00 (.00)	.00 (.00)
Quadratic	Intercepts	.04 (.02)	.03 (.02)	.02 (.01)	.03 (.02)	.02 (.02)	.02 (.01)
	Linear slopes	.01 (.01)	.01 (.01)	.00 (.01)	.01 (.01)	.01 (.01)	.00 (.01)
	Quadratic slopes	.00 (.00)	.00 (.00)	.00 (.00)	.00 (.00)	.00 (.00)	.00 (.00)
Mixed	Intercepts	.05 (.02)	.04 (.02)	.03 (.01)	.03 (.02)	.02 (.01)	.02 (.01)
	Linear slopes	.01 (.01)	.01 (.01)	.01 (.00)	.01 (.00)	.01 (.00)	.01 (.00)
	Quadratic slopes	.00 (.00)	.00 (.00)	.00 (.00)	.00 (.00)	.00 (.00)	.00 (.00)

RMSE was computed for 6, 10, and 20 coefficients of the intercepts, with linear and quadratic slopes depending on the simulation condition

### Coverage and overlap

Table 5 presents the results regarding coverage of the standard errors of the random effects as well as the overlap of the empirical-Bayes credible intervals of the predictions of the levels of random effects. Coverage was computed as the proportion of replications where the true value of the levels of random effects was included in the 95% empirical-Bayes credible intervals constructed by the standard errors of the random effects. Overlap was computed as the proportion of comparisons of empirical-Bayes credible intervals around the levels of random effects that overlap as a measure of the distinctiveness of their 95% empirical-Bayes credible intervals. The proportions were computed for 6, 10, and 20 variables in each replication, depending on the simulation condition. The interest in the interpretation of these standard errors is mainly focused on the linear and quadratic slopes.

**Table 5:** Mean coverage of standard errors of random effects of variables, and mean overlap between empirical-Bayes credible intervals

Indicator	Trajectories	No. individuals	200			500		
		No. variables	6	10	20	6	10	20
Mean coverage	Linear	Intercepts	.94	.98	.99	.95	.98	.99
		Linear slopes	1.00	1.00	1.00	1.00	1.00	1.00
	Quadratic	Intercepts	.84	.90	.95	.87	.92	.96
		Linear slopes	.92	.96	.99	.94	.97	.99
		Quadratic slopes	.98	.99	1.00	.98	.99	1.00
	Mixed	Intercepts	.77	.85	.91	.80	.87	.94
		Linear slopes	.92	.96	.98	.93	.97	.99
		Quadratic slopes	.33	.21	.18	.33	.20	.14
Mean overlap	Linear	Intercepts	.01	.04	.12	.00	.01	.06
		Linear slopes	.00	.00	.10	.00	.00	.04
	Quadratic	Intercepts	.05	.10	.16	.07	.09	.12
		Linear slopes	.02	.05	.12	.02	.04	.07
		Quadratic slopes	.01	.03	.11	.00	.02	.05
	Mixed	Intercepts	.03	.12	.16	.01	.03	.10
		Linear slopes	.01	.09	.14	.00	.01	.09
		Quadratic slopes	.33	.40	.45	.33	.40	.45

Coverage was computed as the proportion of replications where the true simulated parameter of the levels of random effects was included in the 95% empirical-Bayes credible interval constructed by the standard errors of the random effects. Overlap was computed as the mean of the proportion of the 95% empirical-Bayes credible intervals of variables that overlap. The proportions were computed for 6, 10, and 20 variables in each replication

Coverage results show that the standard errors would be overestimated in scenarios with linear trajectories, regardless of conditions of individuals and variables. In the case of quadratic trajectories, there is a tendency to overestimate the standard errors when larger sample sizes are considered. The same applies to the linear effects in conditions of mixed trajectories, although the coverage of the standard errors of the quadratic slopes is very small (this latter result was expected given that half of the variables present a quadratic slope, whereas the other half do not). Overall, these results indicate that larger sample sizes for individuals and variables increase variability, which results in larger estimations of the standard errors of the random effects. Moreover, these results could cause larger type II errors, leading one to conclude that there are no statistically significant differences for some levels of the random effects, although the expectation was to find shorter intervals. However, it would be unlikely to find type I errors, because these standard error estimations are conservative.

Overlap results indicate that the empirical-Bayes credible intervals of the levels of the random effects are precise and distinctive. That is, the proportion of empirical-Bayes credible intervals that overlap is relatively small, including those conditions where coverage was large, thus preserving their distinctiveness. It is worth mentioning the different effects of individuals and variables. Larger sample sizes lead to less overlapping between the empirical-Bayes credible intervals than smaller sample sizes, probably because there is greater statistical power in the former conditions. Conversely, an inverse relation is found between the number of variables and the mean overlap. We believe that this result can be explained by the greater difficulty for conditions with more variables because there are more levels of the random effect under the same metric range. Finally, a large overlap result was observed for the quadratic slopes in mixed trajectories. This was the result of half of the variables presenting a quadratic slope, but not the other half.

### Forecasting individual trajectories from MEM-CR predictions

In order to evaluate the model performance in forecasting individual trajectories, we evaluated two different versions of RMSE for observed and unobserved individual scores. The first version implies three individual observed scores per variable, while the second one implies 12 unobserved scores. Table 6 presents the resulting RMSE values. The lowest value of this measure is zero (if the model predictions were exactly the same as the simulated trajectories) and there is no maximum value, although it can be interpreted in the original metric of the dependent variable. To interpret RMSE values, we must consider that we simulated a standard deviation of measurement error of .50, which is a completely random error variance for each individual *i* in variable *v* at time *t* (see simulated parameters in Table 2). The RMSE values of the difference between the simulated trajectories and the predicted scores of individuals for observed (*n* = 3) time points in this cohort-sequential design can then be interpreted as very accurate model predictions of individual scores, with RMSE even slightly smaller than the measurement error. Additionally, we need to consider that the simulated random variance is equal to 1.175 for individuals and.483 for variables (with small differences among the different number of variables), which together with the random error variance constitutes a total empirical variance of 2.155. As a result, RMSE values of 2.155 would imply that the error predictions for individuals in the variable-specific trajectories are equal to the simulated variability for individuals and variables. Thus, RMSE results for unobserved individual scores are fairly small in all the simulation conditions compared to the simulated total empirical variance, and they are relatively close to the simulated measurement error.

**Table 6:** Means (and standard deviations) of the root mean square error (RMSE) of the difference between the simulated trajectories and the predicted scores of individuals for observed (*n* = 3) and unobserved (*n* = 12) time points of the simulated cohort-sequential design

Trajectories	No. individuals	200			500		
	No. variables	6	10	20	6	10	20
Linear	Observed	.48 (.01)	.49 (.00)	.47 (.01)	.47 (.01)	.49 (.01)	.48 (.01)
	Unobserved	.58 (.01)	.55 (.01)	.59 (.02)	.59 (.01)	.59 (.02)	.57 (.03)
Quadratic	Observed	.48 (.01)	.49 (.01)	.48 (.01)	.47 (.01)	.48 (.01)	.47 (.01)
	Unobserved	.72 (.03)	.68 (.02)	.75 (.04)	.73 (.03)	.69 (.03)	.71 (.04)
Mixed	Observed	.48 (.01)	.49 (.00)	.47 (.01)	.47 (.01)	.48 (.01)	.48 (.01)
	Unobserved	.72 (.03)	.68 (.02)	.75 (.04)	.73 (.03)	.68 (.03)	.71 (.04)

Given the simulated cohort-sequential design, there are three observed scores per individual and variable, and 12 unobserved per individual and variable

**Fig. 3:**
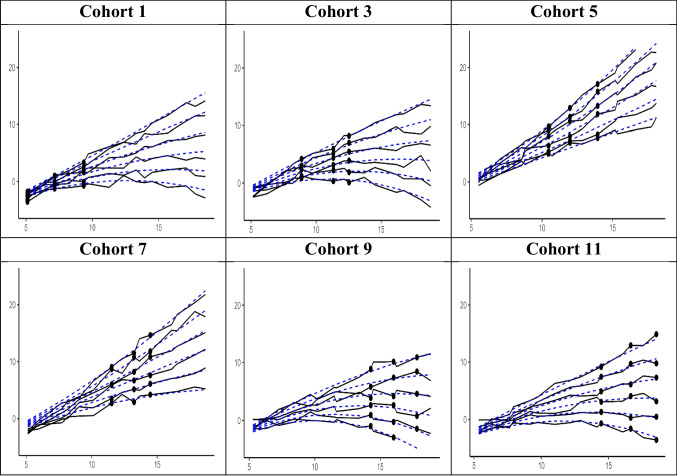
Forecasting quadratic variable-specific trajectories for individuals from different cohorts. *Note*. Dots represent the measurement of the variables. Black continuous lines represent the simulated true trajectory for each variable. Blue discontinuous lines represent the predicted/forecasted trajectory for each variable. Forecasting from a dataset with 200 individuals and six variables

Figure 3 presents the forecasted quadratic trajectories of six variables for individuals of six different cohorts. This plot shows that the predicted trajectories are accurate, regardless of the selected cohort. In other words, the MEM-CR can adequately recover the true simulated trajectories both backward and forward using just three observations per variable. Additionally, this figure also shows the large variability across individuals and variables for the intercepts and both linear and quadratic slopes. This variability is particularly apparent in individuals and variables that present larger changes over time (in some cases, changes are more linear, while other cases present larger quadratic effects), relative to other individuals or variables whose changes are less pronounced.

Additionally, we computed mean bias for the different cohorts and levels of the random-effect variables to evaluate potential shrinkage effects when forecasting trajectories for specific individuals. Thus, bias was computed only for the 12 unobserved (but forecasted) individual scores of the trajectory. Supplementary Table 1 presents the bias for the 1,000 simulated datasets with six variables and 200 individuals, comparing the predicted scores with the simulated true trajectories. Results show a lack of shrinkage effects, as the bias of the predictions of variables with higher or lower predictions did not present a tendency to overestimate or underestimate the individual scores for the trajectories. Moreover, no relevant cohort effects were observed. This indicates that the error resulting from the model is relatively small, does not follow any specific trend (e.g., shrinkage), and is similar when forecasting past or future states. A similar pattern of results was found across the other simulation conditions.

## Empirical illustration

**Fig. 4:**
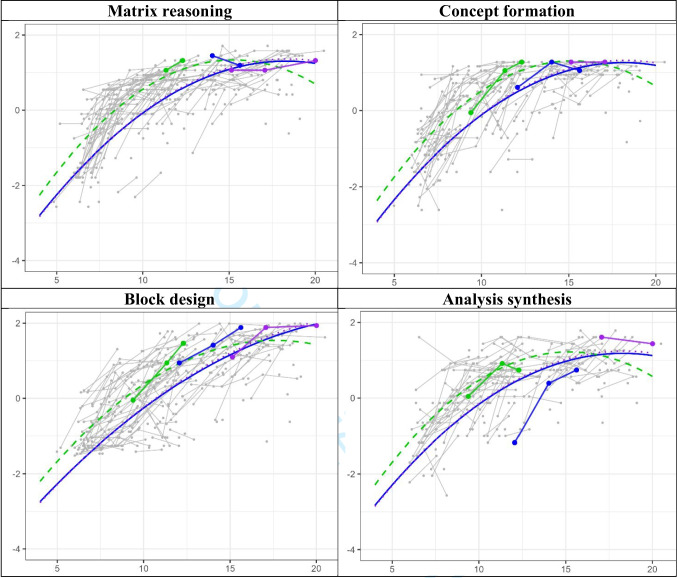
Examples of predicted trajectories of three individuals from different cohorts in the four measures of fluid reasoning in the NORA study. *Note*. Gray lines are the observed trajectories of all the individuals of the sample. Short colored lines with dots represent observations of three randomly selected individuals. Long colored lines represent the predicted trajectory for those individuals. Colors (blue, green, and purple) represent the individual

To illustrate the implementation of the model as well as to provide an interpretation of its parameter estimates, we analyzed data from the NORA (Neural Development of Reasoning Ability) study (Ferrer et al., 2009; Wendelken et al., 2011). This study aimed to investigate the development of fluid reasoning from childhood to adolescence using a cohort sequential design with up to three measurement occasions (*n*_1_ = 201, *n*_2_ = 122, *n*_3_ = 70) and intervals of 12–24 months. Approximately half of the participants were women, and the age at the first measurement occasion ranged from 4.83 to 19.1 years. Fluid reasoning is the ability to use logic and solve problems in new situations and is presumably independent of previously acquired knowledge (Cattell, 1987). To assess fluid reasoning, the study involved four measures: block design (BD) and matrix reasoning (MR), which are subtests of the Wechsler Abbreviated Scale of Intelligence (WISC-R; Wechsler, 1981), and analysis synthesis (AS) and concept formation (CF), which are subtests of the Woodcock–Johnson Tests of Achievement (WJ-R; Woodcock et al., 1990). Descriptive analysis of these variables can be found in Table 7 (see also Fig. 4 for a graphical representation of the data).

**Table 7:** Means (and standard deviations) of the fluid reasoning measures from the NORA study

	Matrix reasoning	Block design	Concept formation	Analysis synthesis
Time 1 Time 2 Time 3	21.8 (7.71) 26.3 (6.47) 28.7 (4.08)	32.0 (19.1) 40.5 (19.9) 49.0 (16.8)	23.5 (9.01) 27.9 (7.54) 31.9 (4.47)	24.7 (5.74) 27.0 (4.59) 29.4 (3.20)

When implemented with these data, the MEM-CR model revealed similar developmental trends to those from the simulation, which was based on a completely different empirical study (Martínez-Huertas & Ferrer, 2023). Table 8 presents these results. Given that the variables were standardized according to the first measurement occasion, the results indicate a negative intercept (*b* = − 4.96, *SE* =.368, *p* <.001) representing the overall fluid reasoning levels at *t* = 0, a large positive linear effect of age (*b* =.717, *SE* =.235, *p* <.01), and a smaller and nonsignificant negative quadratic effect (*b* = −.021, *SE* =.241, *p* =.930). These parameter estimates mean that the general developmental trajectory (common to all individuals and variables of the study) of fluid reasoning has a large positive linear effect of age and a smaller negative quadratic effect. The lack of statistical significance of the quadratic effect reflects the fact that the four variables differ in their developmental trends. Additionally, the correlations between the fixed effects were small: *r* = −.148 between the intercept and the linear effect, *r* =.011 between the intercept and the quadratic effect, and *r* = 0.004 between the linear and the quadratic effects. This suggests that the individual rates of change are not determined by their initial levels (intercepts). The variance components of the model reveal important differences between individuals and across variables in both the starting points and rates of change. Finally, the residual variance was.487, which represents unexplained variability after accounting for both individual and variable differences.

**Table 8:** Parameter estimates for the NORA study

Fixed effects	Estimate	*SE*	*t*-statistic	*p*-value
${\beta}_{0}$	− 4.961	.368	− 13.481	<.001
${\beta}_{1}$	.717	.235	3.055	<.01
${\beta}_{2}$	−.021	.241	−.089	.930
${r}_{{\beta}_{0},{\beta}_{1}}$	−.148			
${r}_{{\beta}_{0},{\beta}_{2}}$	.011			
${r}_{{\beta}_{1},{\beta}_{2}}$	.004			
Random effects (*SD*s)				
Individuals	σ_0i_	.469		
	σ_1i_	.130		
	σ_2i_	.008		
Variables	σ_0v_	.479		
	σ_1v_	.460		
	σ_2v_	.483		
Error	*e*_ivt_	.487		
Variable-specific predictions	Intercepts [95% E-BCI]		Linear effects [95% E-BCI]	Quadratic effects [95% E-BCI]
MR	− 5.059 [− 5.595 to − 4.524]		.754 [.660 to.848]	−.023 [−.027 to −.019]
BD	− 5.037 [− 5.048 to − 3.973]		.599 [.505 to.693]	−.015 [−.019 to −.011]
AS	− 5.037 [− 5.643 to − 4.430]		.739 [.636 to.843]	−.023 [−.027 to −.019]
CF	− 5.236 [− 5.804 to − 4.668]		.776 [.675 to.877]	−.024 [−.028 to −.020]
		Model fit		
		Deviance = 2,466.606		
		AIC = 2,498.606		
		BIC = 2,582.353		

*SE* standard error; *SD* standard deviation; *MR* matrix reasoning; *BD* block design; *CF* concept formation; *AS* analysis synthesis; *E-BCI* empirical-Bayes credible intervals. ** *p* <.01. *∑*_*A*_ and *∑*_*B*_ were estimated but not reported due to space limitations. Intercepts and slopes of variables are the result of the combination of the fixed effects and the variance components (random effects) for each variable

The variance estimates of the model for the four variables indicate that there are relevant differences between the variables of the study regarding the development of fluid reasoning (see “Random effects” subsection of Table 8). These estimates show that all the variables follow a similar trend, but with differences among them. The initial levels vary slightly between variables, with CF showing the lowest initial values and BD showing slightly higher starting points. The magnitude of the linear effect of age also differs across variables, with CF presenting the largest rate of change and BD the lowest. Similarly, the variables reveal a negative quadratic effect, indicating a decelerated rate of change, as well as some differences between them, with BF showing a larger curvature and CF a slower one. These results suggest that some variables are more sensitive to developmental change than others. Even with these differences among variables, it is possible to see a common developmental trajectory of these individuals among the four variables. In other words, their changes may have a common underlying construct. Finally, based on this model, we forecasted variable-specific trajectories for individuals. Figure 4 presents such predicted variable-specific trajectories for three individuals from the NORA study. These individuals show differences in their trajectories regarding the time of the available information and the predicted curvature; that is, they differ in the initial levels and the linear and quadratic rates of change.

## Discussion

### Summary of findings

We present analyses examining the performance of the MEM-CR in capturing within- and between-variability across individuals and variables in multivariate longitudinal data. We tested this model in a cohort-sequential design using a continuous-time metric. Such design aims to describe the complete longitudinal trajectory of individuals over a wide time range with a small number of observations per individual. Moreover, an important aim in multivariate longitudinal data analysis is to describe the individual trajectories for multiple variables, capturing their common and idiosyncratic variability. Our findings support the use of MEMs-CR to estimate general and variable-specific trajectories in cohort-sequential designs.

First, we found that the estimations of MEMs-CR can accurately describe general trajectories (common to all individuals and variables) as well as their variability across individuals and variables. We observed a lack of bias in the estimation of the fixed effects and the variability of individuals regardless of the complexity of the trajectories. In contrast, we found a slightly larger percentage of bias for the estimation of the variability of the variables for quadratic trajectories only (not for linear or mixed trajectories) for larger sample sizes. Specifically, these variances were slightly overestimated, meaning that the estimated variability of the trajectories within variables is slightly larger than the true simulated value. However, as we describe next, this was not a limitation when predicting the true simulated values of specific levels of such random effects, nor when forecasting the complete trajectories.

Second, our findings show that MEMs-CR can predict the trends in variable-specific trajectories regardless of the different conditions of the study. That is, the model adequately recovered the estimations of the levels of the random factors. This means that the trajectories of specific variables captured by a cluster or random effect were adequately predicted, even when considering effects with very close (e.g., random quadratic slopes) or very different (e.g., random intercepts) levels of random effects.

Third, the results regarding coverage showed that the estimated standard errors of the time effects within random effects are generally overestimated. That is, the intervals were greater than the nominal coverage probability with a 95% confidence. Along these lines, there was a tendency to overestimate standard errors of random effects when the sample size was larger (especially for a larger number of variables). These results can translate into larger type II errors, so one would conclude no statistically significant differences for some levels of the random effects. In other words, it would be very unlikely to find type I errors with standard error estimations being conservative. Nevertheless, the empirical-Bayes credible intervals of the levels of the variables were generally precise and allowed us to differentiate between variable-specific trends. Besides this general trend, some differences across the simulated conditions of the trajectories are worth mentioning. Conditions with linear trajectories showed the largest overestimation of the standard errors of linear slopes but less overlap regarding their empirical-Bayes credible intervals, while conditions with quadratic and mixed trajectories showed more variability surrounding the nominal coverage probability for linear slopes and slightly larger overlap. Moreover, as expected, the estimation of the standard errors of the quadratic effects in conditions with mixed trajectories (where some variables have quadratic and others have linear effects) was far from the nominal coverage probability, likely leading to biased standard errors and large overlaps between the empirical-Bayes credible intervals of the variables.

Fourth, we found that the MEM-CR could adequately predict individual and variable-specific trajectories. This finding has important implications, as the individual and variable-specific predictions of the model can forecast trajectories for the complete age range under study (here, 15 years) from just a few observations per individual. This implies that researchers could forecast both past and future states of multivariate states or specific variables to make individual decisions. Moreover, no shrinkage was found for variables with different levels (i.e., higher intercepts and time effects vs. small intercepts and time effects), probably related to the different sources of variability that are considered in these models (i.e., this might be expected to be different for univariate longitudinal analyses). It is worth noting that the capacity of the model to correctly extrapolate values outside of the time range of individual observations to infer both past and future states benefited from a lack of model misspecification in the simulation study. This means that the trajectories were adequately predicted when the data followed the simulated trend, but real-world applications always have some degree of misspecification or uncertainty. The good accuracy of the forecasting of multivariate longitudinal trajectories reflects an “ideal” correctly specified setting, given that the predictive model matched the data-generating process. Thus, these results should be understood as an upper bound on performance. In real-world applications, the quality of predictions outside the observed time range will likely be worse. Additional robustness checks, such as out-of-sample validation, may be helpful to evaluate the real accuracy of the model predictions.

We also showed the use of the model in an empirical illustration regarding cognitive development from a cohort-sequential design (Ferrer et al., 2009; Wendelken et al., 2011). In these empirical analyses we illustrated how the fixed effects describe the general trajectory of fluid reasoning (common to all individuals and variables), that the random effects show how the parameters of those trajectories differ across individuals and variables, that some variables are more sensitive to developmental changes than others, and that it is possible to forecast variable-specific trajectories for individuals with their own idiosyncratic trajectories.

### Theoretical and methodological considerations

In general, our findings are generalizable to the different simulation conditions that ranged from a small number of variables to other conditions with multiple indicators of a single variable. Thus, these conclusions are useful for similar designs of multivariate longitudinal studies with a few total scores of variables that share a common developmental process and other datasets with multiple indicators (i.e., items) of a specific variable (see Donnellan et al., 2023, for the latter scenario). Both would also be related to the assumption of an underlying common construct that explains the variability in the different indicators. Fixed effects are just the averaged effect of the levels of random effects as in composite models, and the levels of the random effects are computed around a specific time effect common to all the variables as in a common factor model. Of course, we must assume that the sampled individuals and variables (or indicators) are representative of the developmental process and psychological construct under study. This means that this analytical approach should only be applied to sets of variables that are meant to measure the same underlying construct. Otherwise, assuming a general trajectory would not be reasonable. Similarly, it is assumed that the changes over time across the variables tend to covary. In other words, the exchangeability principle holds for the different levels of the random effects for both individuals and variables (e.g., Draper et al., 1993; Greenland & Draper, 2005; Hoffman, 2015; Lindley & Smith, 1972; Raudenbush, 1993). This assumption allows one to study the general trend, common to all individuals and variables, but also the heterogeneity of psychological processes, bringing us closer to an idiographic science (see Molenaar, 2004).

The point estimations of the fixed and random parameters and the levels of the random effects of variables were accurately estimated even with large proportions of planned missing data, as in cohort-sequential designs. Some exceptions were found for the variability in the trajectories within variables, which was slightly overestimated for larger sample sizes. This may be interpreted as an inaccurate partitioning of the empirical variability as a result of having only three available observations per variable and individual, thus adding extra measurement error, which would lead to overestimated variability. Moreover, the clusters were unbalanced, with a relatively small number of variables, which is inherent to multivariate data and reduces the model performance. We believe that a direct consequence of such an issue is greater uncertainty in the predictions of the coefficients of the random effects. As a result, the standard errors of the levels of the random effects were generally overestimated and more conservative than expected. In any case, overlap results showed that they are useful and generally precise for differentiating between the predictions of variable-specific trajectories.

Even more importantly, our findings suggest that the MEM-CR allows one to adequately infer the complete trajectories of specific individuals and variables. This finding implies that these models can balance the idiosyncratic variability in both individuals and variables around their intercepts and the slopes of time effects under conditions of trajectories of different complexity. Thus, these models are useful for predicting past and future unobserved individual states in multivariate longitudinal data. This is particularly useful in cohort-sequential designs, in which there is a small proportion of available observations per individual and variable, but we are interested in studying the complete longitudinal trajectory. Given these results, we can also confirm that it is possible to unbiasedly recover the parameters that define the developmental processes under study in cohort-sequential designs using mixed-effects models with crossed random effects and the variability within them, as well as in other statistical models like latent growth curves (Duncan et al., 1996) or latent change score models (Estrada & Ferrer, 2019; Martínez-Huertas et al., 2024). In fact, recent research has shown that it is possible to accurately forecast individual scores in very similar cohort-sequential designs for univariate systems using an extension of the Kalman filter scores (Martínez-Huertas et al., 2024). In comparison, the MEM-CR provides researchers with an accessible but powerful extension for the analysis of multivariate longitudinal data, which is, at least nowadays, a complicated issue due to the complexity of other formal alternatives.

In relation to the various simulated conditions for BLUPs, our findings suggest that the standard errors associated with the quadratic effect of mixed trajectories exhibited a propensity for bias. These simulation conditions were characterized by the same number of levels of the random factor with either the same or different sign as the fixed effect. Apart from finding effects that are not generalizable and would likely lack statistical significance, the reliability of the inferences drawn from the standard errors, and their empirical-Bayes credible intervals, could be compromised. Therefore, under these conditions, we advise researchers to be cautious when interpreting differences between the levels of a cluster (i.e., individuals or variables). Nevertheless, the remaining model predictions were accurate under conditions with mixed trajectories, with only those quadratic effects being adversely affected by this BLUP condition.

### Limitations and future directions

Several limitations are worth mentioning. First, we simulated some specific parameters based on previous analyses of a US cohort-sequential design, namely, the NLSY79 data (Center for Human Resource Research, 2009; Chase-Lansdale et al., 1991). It will be important to examine the robustness of the MEM-CR with other studies that include different time effects and different variability. Our results showed that the random effects differed between the clusters and that the fixed effects were correctly estimated. Given these findings, we believe that future analyses using different parameters are likely to yield comparable or superior results. This conclusion stems from our methodology, which involved the simulation of asymmetric variances in clusters with small sample sizes, a very demanding condition for the estimation of random effects (Martínez-Huertas & Olmos, 2022).

Second, we simulated a representative cohort-sequential design where individuals were measured in three nonconsecutive years. However, the characteristics of the data sampling could significantly influence model performance, as they determine the sparsity of the data. It will be important to evaluate the consequences of different ratios between observed and unobserved scores of individual-variable pairs. We would expect better results in scenarios with more information and less uncertainty about the trajectories. Future research should also examine whether these findings extend to other types of longitudinal designs with continuous-time metric, such as intensive longitudinal studies (e.g., Castro-Alvarez et al., 2022; Hamaker & Muthén, 2020; Hamaker & Wichers, 2017; Martínez-Huertas & Ferrer, 2026; McNeish et al., 2021). Some advances are being made in developing Bayesian nonlinear models with crossed random effects (e.g., Rohloff et al., 2024), but modeling more complex time effects such as nonlinear oscillations is challenging for mixed-effects models. Such challenges probably require a different estimation method to achieve adequate modeling and forecasting, such as applying a filtering approach (e.g.,Hunter et al., 2022; Ou et al., 2023). In this case, we have focused on a particular multivariate growth curve based on previous research, but it is possible to extend the rationale presented in this paper to nonlinear mixed-effects models that could handle the nonlinear form and the degree of nonlinearity more naturally (e.g., Lindstrom & Bates, 1990). Future research should analyze whether nonlinear mixed-effects models adequately recover the population parameters within cohort-sequential designs and assess the performance of their random effects and their standard errors.

Third, we did not control the correlations between the different fixed and random effects of the model. Along these lines, it would be important to manipulate the error term of the different variables to evaluate the impact on the residual variance and the other parameters of the model, as not all the variables tend to present exactly the same reliability over time. This is a topic of special interest in similar contexts, for example, with intensive longitudinal data in dynamic structural equation modeling (McNeish et al., 2021). Moreover, we have focused on random effects that are sampled from a normal distribution, but the performance of the random effects could be worse if the true distribution is far from normal (Hui et al., 2021). Future studies should analyze the influence of the shrinkage and the robustness of the predictions of these models with respect to deviations from the normal distribution in the random effects.

Fourth, we evaluated time effects using time as a time-varying covariate with a continuous-time metric in which zero represents the initial state (*t* = 0) of the individual in all the variables and each time unit represents 1 year. We are aware of the relevance of centering the predictors in multilevel modeling (e.g., Enders & Tofighi, 2007; Hamaker & Muthén, 2020; Raudenbush & Bryk, 2002; Wu & Wooldridge, 2005; Yaremych et al., 2023). While we believe that cohort-sequential designs require the use of that specific time metric to adequately describe trajectories, it will be important to evaluate how centered covariates interact with time and to determine the consequences of not centering.

Finally, and more importantly, the estimations of the MEMs-CR studied in this paper are the result of polynomial equations. While these models are a relatively simple solution for studying multivariate longitudinal trajectories, they can also lead to some polynomial artifacts in particular cases. For example, one of the predictions in Fig. 4 presents how a quadratic effect that may fit the initial development during early childhood can be mathematically forced to present a negative slope, decreasing the cognitive levels at later ages. Although that decline may be theoretically plausible, it is probably an artifact generated by the limitations of polynomials. For this reason, some authors have suggested either flexible smoothers (see, for example, the R *mgcv* package, Wood, 2015, 2017) or using theory-motivated nonlinear functions with interpretable parameters (e.g., Jenss–Bayley/exponential asymptotic forms; see for example Grimm & Ram, 2009; Pinheiro & Bates, 2000; Ram & Grimm, 2007; Ratkowsky, 1989). A comprehensive study of these techniques is beyond the scope of our study. However, we advise users of these models to interpret potential polynomial artifacts with caution.

## Conclusions

In this study, we showed that the MEM-CR can be used to estimate within- and between-variability of multivariate trajectories. We showed that (a) this model can estimate the general trajectories (common to all individuals and variables) and their variability, plus the variable-specific trajectories through the predictions of the levels of the random factors; (b) the estimations of the standard errors of random effects are very important for making decisions for specific variables, although they may be overestimated (it would be very unlikely to find type I errors, being conservative); and (c) the model predictions can adequately forecast individual and variable-specific trajectories from just a few observations per individual. This last conclusion is particularly important for researchers, making it possible to obtain complete individual multivariate trajectories throughout a wider age range than the period covered by the measurement occasions. Thus, we recommend researchers use this statistical model to study within- and between-variability of multivariate trajectories in longitudinal cohort-sequential designs.

## Supplementary Information

Below is the link to the electronic supplementary material.

Supplementary file1 (PDF 73 kb)

## Data Availability

Simulated data and code analysis are available as supplementary materials to illustrate the use of this model. Empirical data are not publicly available but are available from the authors on reasonable request. https://osf.io/agcx6/.

## Footnotes

1 Note that this particular model and simulation study are used to accommodate multivariate growth curves within cohort-sequential designs using a continuous time metric. But the MEM-CR for individuals and variables can be used for the analysis of other multivariate longitudinal designs as well (Martínez-Huertas & Ferrer, 2023).

2 This model can also be used with unstandardized variables. In that scenario, however, the interpretability of the model estimates requires caution because they mix the target developmental effects and the differences in the metrics of the variables.

3 The number of comparisons was defined as the number of upper-triangle off-diagonal entries in a matrix of 6 × 6 and a matrix of 20 × 20.

## Code availability

R code is available as supplementary materials to illustrate the use of this model. https://osf.io/agcx6/.

## Declarations

### Open practices statement

The study was not preregistered. Simulated data and analysis code are available as supplementary materials to illustrate the use of this model. Empirical data are not publicly available but are available from the authors on reasonable request.
