# Supplementary material for: Estimating multivariate longitudinal trajectories using mixed-effects models with crossed random effects
Source: Behav Res Methods. 2026 Jun 17;58(7):201. doi: 10.3758/s13428-026-03070-5 (PMC13275786; doi:10.3758/s13428-026-03070-5)
Supplement: Supplementary file 1 — Supplementary file1 (PDF 73 kb) [file 13428_2026_3070_MOESM1_ESM.pdf]

SUPPLEMENTARY MATERIALS

Estimating multivariate longitudinal trajectories using mixed-effects models with crossed random effects

Supplementary Table 1. Mean bias of the predicted scores for specific individuals and variables in the unobserved (*n*=12) time points in simulated data sets (6 variables and 200 individuals) comparing with the simulated true latent trajectory.

**Trajectories**	**Levels**	**Cohort**										
		**1**	**2**	**3**	**4**	**5**	**6**	**7**	**8**	**9**	**10**	**11**
Linear	Higher	.012	.001	-.008	.013	-.003	-.008	-.008	-.005	.007	.002	-.002
	Medium-Higher	.008	.006	-.005	.006	-.001	.000	.001	-.004	.001	.000	-.004
	Medium	.009	.004	-.003	.011	.001	-.006	.000	.001	.007	.003	.000
	Medium-Lower	.009	.006	-.006	.015	-.002	-.009	-.005	-.006	.006	.001	-.001
	Lower	.006	.001	-.005	.005	-.005	-.005	.003	-.002	.006	.000	-.004
Quadratic	Higher	-.008	.001	-.023	.028	-.007	-.015	.016	.001	-.007	-.019	.005
	Medium-Higher	-.003	.002	-.014	.009	.000	-.007	.005	.002	-.007	-.009	.001
	Medium	-.007	.007	-.017	.016	-.002	-.012	.014	.002	-.005	-.013	.005
	Medium-Lower	-.008	.000	-.027	.024	-.005	-.016	.019	.000	-.007	-.018	.005
	Lower	.001	.001	-.009	.006	-.004	-.007	.012	-.001	-.002	-.010	.004
Mixed	Higher	-.009	-.002	-.026	.023	-.011	-.017	.012	-.001	-.009	-.023	.002
	Medium-Higher	-.002	.001	-.015	.008	-.001	-.008	.004	.001	-.008	-.010	.000
	Medium	-.004	.000	-.014	.017	.000	-.009	.015	.005	-.001	-.011	.008
	Medium-Lower	-.007	-.001	-.028	.021	-.007	-.015	.017	.000	-.007	-.019	.004
	Lower	.000	-.001	-.010	.003	-.006	-.008	.010	-.002	-.004	-.012	.002

Note. Variables were divided into five groups depending on the point estimations of their random effects. “Low levels” are variables with intercepts of -2.50, linear slopes of .75 and quadratic effects of -.05 (except for linear trajectories that did not present quadratic effects). “High levels” are variables with intercepts of -1.50, linear slopes of 1.25 and quadratic effects of .00. The other categories range from one high to low levels.

For Review Only
